# Exercise combined with postbiotics treatment results in synergistic improvement of mitochondrial function in the brain of male transgenic mice for Alzheimer’s disease

**DOI:** 10.1186/s12868-023-00836-x

**Published:** 2023-12-18

**Authors:** Attila Kolonics, Zoltán Bori, Ferenc Torma, Dora Abraham, János Fehér, Zsolt Radak

**Affiliations:** 1 Research Centre for Molecular Exercise Science, Hungarian University of Sport Science, Alkotas str. 44, Budapest, 1123 Hungary; 2 Ophthalmology Unit, NESMOS Department, Faculty of Medicine and Psychology, Sant’Andrea Hospital, ‘‘Sapienza’’ University of Rome, Rome, Italy; 3 Sports Neuroscience Division, Advanced Research Initiative for Human High Performance (ARIHHP), Faculty of Health and Sport Sciences, University of Tsukuba, Tsukuba, Ibaraki 305-8574 Japan; 4 Laboratory of Exercise Biochemistry and Neuroendocrinology, Faculty of Health and Sport Sciences, University of Tsukuba, Tsukuba, Ibaraki 305-8574 Japan

**Keywords:** *Bifidobacterium longum*, *Lactobacillus acidophilus*, Alzheimer’s disease, Cognitive function, Gut microbiota, Mitochondrial protein quality control, Metal ion chelation

## Abstract

**Background:**

It has been suggested that exercise training and postbiotic supplement could decelerate the progress of functional and biochemical deterioration in double transgenic mice overexpresses mutated forms of the genes for human amyloid precursor protein (APP^sw^) and presenilin 1 (m146L) (APP/PS1^TG^). Our earlier published data indicated that the mice performed better than controls on the Morris Maze Test parallel with decreased occurrence of amyloid-β plaques in the hippocampus. We investigated the neuroprotective and therapeutic effects of high-intensity training and postbiotic supplementation.

**Methods:**

Thirty-two adult APP/PS1^TG^ mice were randomly divided into four groups: (1) control, (2) high-intensity training (3) postbiotic, (4) combined (training and postbiotic) treatment for 20 weeks. In this study, the whole hemibrain without hippocampus was used to find molecular traits explaining improved brain function. We applied qualitative RT-PCR for gene expression, Western blot for protein level, and Zymography for LONP1 activity. Disaggregation analysis of Aβ-40 was performed in the presence of *Lactobacillus acidophilus* and *Bifidobacterium longum* lysate.

**Results:**

We found that exercise training decreased Alzheimer’s Disease (AD)-related gene expression (NF-kB) that was not affected by postbiotic treatment. The preparation used for postbiotic treatment is composed of tyndallized *Bifidobacterium longum* and *Lactobacillus acidophilus*. Both of the postbiotics effectively disaggregated amyloid-β/Aβ-40 aggregates by chelating Zn^2+^ and Cu^2+^ ions. The postbiotic treatment decreased endogenous human APP^TG^ protein expression and mouse APP gene expression in the hemibrains. In addition, the postbiotic treatment elevated mitochondrial LONP1 activity as well.

**Conclusion:**

Our findings revealed distinct mechanisms behind improved memory performance in the whole brain: while exercise training modulates NF-kB signaling pathway regulating immune response until postbiotic diminishes APP gene expression, disaggregates pre-existing amyloid-β plaques and activates mitochondrial protein quality control in the region of brain out of hippocampus. Using the above treatments complements and efficiently slows down the development of AD.

## Introduction

Alzheimer’s disease (AD) is a currently untreatable disease with a gradually increasing incidence, resulting in mild to very severe memory loss and a variety of comorbidities [1, 2]. In the absence of an effective pharmacological treatment for AD, there is considerable interest in potential means of preventing AD. There is ample evidence that regular physical exercise can attenuate the onset of AD and even slow its progression [3–5]. Exercise has systemic effects on the body and shows positive, health promoting effects in all organs, despite of the very different oxygenation and metabolism of different organs during physical exercise [6]. One of the fascinating adaptive responses to exercise training involves the gut microbiome. The gut–brain axis is thought to significantly affect the function of both organs, and growing body of data suggest that the gut microbiome has a strong influence on the gut–brain axis [7, 8]. A clinical study has shown that *Bifidobacterium bifidum* and *Bifidobacterium longum* containing postbiotics induce changes in the gut microbiota that promote mental flexibility and alleviate stress in healthy older adults, along with causing changes in gut microbiota [9]. The gut microbiome is crucial for the breakdown of dietary nutrients, the regulation of intestinal and systemic immune responses, the production of small molecules critical for metabolism in the gut, and the production of various gases that can affect cellular function. Due to the complex function of the gut microbiome, the diversity of microbes is defined by the number and frequency of distribution of different types of organisms [1].

In our previous work, we reported that on APP/PS1 transgenic mice exercise training and postbiotic treatment decreased the amount of amyloid-β plaques and improved spatial memory assessed by Morris maze test [10]. The result suggests that exercise training and postbiotic treatment have different mechanisms of actions. Our treatment contained tyndallized *Bifidobacterium longum* and *Lactobacillus acidophilus* lysates, which could cause the beneficial effects of our treatment. FRAMELIM® contains previously killed postbiotics, which are called postbiotics according to the new nomenclature [11]. Previously, the intracellular cell-free extract of these strains was shown to have potent Fe^2+^, Cu^2+^ chelating ability [12], which has not been investigated to play a role in modulating the development of AD.

Supplementation with *Lactobacillus acidophilus* has also been shown to attenuate the effects of chronic fatigue and restored T cell function in athletes [13]. The results of another human study suggest that treatment with *Bifidobacterium* and *Lactobacillus* reduces the incidence of upper respiratory tract infections in healthy adults [14]. *Lactobacillus acidophilus* appears to harbour the surface layer proteins with the molecular weight of approximately 45 kDa, the administration of which can suppress myeloperoxidase activity and TNF-α expression [15]. Deletion of the gene encoding lipoteichoic acid (LTA) biosynthesis in *Lactobacillus acidophilus* resulted in down regulation of IL-12 and TNFα and elevation of IL-10 levels and suppressed the ability of T-cell activation [16]. Our previous results showed that treatment with *Bifidobacterium* and *Lactobacillus* treatment decreased Aβ levels [10], and similar data have been reported by other works as well [17, 18]. These results encouraged us to investigate the molecular mechanism behind this attractive effect of FRAMELIM® treatment on the progression of AD in the transgenic mouse model.

## Materials and methods

### Materials

FRAMELIM® contains tyndallized *Bifidobacterium longum* and *Lactobacillus acidophilus* lysates, in addition vitamins A, B1, B3, B6, B9, B12 and omega 3 fatty acids in cod liver oil was included. SIGMAFAST™ protease inhibitor cocktail tablets and PhosSTOP™ phosphatase inhibitor tablets, Human Aβ40, purity ≥ 90% and Thioflavin T was purchased from Sigma Aldrich. Ltd. (Germany). 2-cyano-3,12-dioxo-oleana-1,9(11)-dien-28-oic acid (CDDO), purity ≥ 95% was from Cayman Chemicals (US). Amyloid beta precursor protein (APP) rabbit (Abcam-ab101492), human reactive APP mouse (Biolegend-6E10 clone), mitochondrial matrix LON peptidase 1 (LONP1) mouse (Proteintech-66043-1-Ig), Succinate dehydrogenase complex flavoprotein subunit A (SDHA) rabbit (SantaCruz-sc-98253), NADH:ubiquinone oxidoreductase core subunit V1 (NDUFV1) mouse (SantaCruz-sc-100566), Glyceraldehyde 3-phosphate dehydrogenase (GAPDH) mouse (Sigma-Aldrich) antibodies was bought from commercial vendors. mAPP, mNF-kB, mSOD-1 primer pairs was ordered from Sigma Aldrich (Germany).

### Experimental animals, grouping, training procedure, and postbiotic supplementation

Thirty two male APP/PS1 transgenic mice (B6C3-Tg (APPswe, PSEN1dE9)85Dbo/Mmjax; APP/PS1^TG^) were randomly assigned to control (APP/PS1^TG^-C), exercise (APP/PS1^TG^-Ex), postbiotic treated (APP/PS1^TG^-Pt) and combined (exercise and postbiotic treated) (APP/PS1^TG^-Ex-Pt) groups. For The open field test, The Morris Water Maze Test, the spontaneous alteration tests, and the microbiome investigation, we added eight wild type mice of similar ages, from the same colony, as absolute controls (Wt). All animals were maintained under controlled conditions of 12-h light/12-h dark cycle, at 25 °C, received unrestricted water and a standard laboratory diet (Special Diet Service Limited, UK). Treatments were carried out for 20 weeks starting at the age of 3 months, with the aim of decreasing the progress of AD development and functional impairment. Interval treadmill running was applied as the exercise regimen for the combined groups. Animals were acclimated to running on a motor-driven treadmill (Columbus Inst. Columbus Ohio) for 2 weeks by gradually increasing the speed to the assigned running speed. Training was performed four times per week for 60 min over a 20-week period. Initially, the high intensity running speed was set at 16 m/min and gradually increased every third week by 1 m/min to 20 m/min. Each individual training session consisted of ten cycles, with each cycle including a 4-min high intensity running session (20 m/min) and a 2-min low intensity running session (10 m/min). All training sessions were performed during the light phase between 8:00 and 11:00 am. The transgenic control group and the postbiotic treated group (APP/PS1TG-C and Pt) were placed on the treadmill and remained there for 5 min/day on the stationary belt. FRAMELIM® was administered five times weekly (120 mg/day) for 20 weeks along with rodent chow (SDS/VRF1(P)). Considering that mice have a high capacity to store vitamin A and B and require a long time (up to 40 weeks) [19] to induce Vitamin A and B deficiency, FRAMELIM® has no significant effect on increasing vitamin levels that may affect amyloidosis. We monitored the food intake of all mice daily, and postbiotic treatment did not alter the amount of food and water the animals consumed. After the 20-week treatment, the animals underwent cognitive testing for 2 weeks, but postbiotic treatments continued even during testing.

### Collection of samples

At the end of the treatment, mice were anaesthetised by (ip) injection of a cocktail (0.1 ml/20 g) of 0.5 g/kg body weight ketamine (Richter) and 0.1 g/kg body weight xylazine (Produlab Pharma). After perfusion with heparinised ice-cold physiological saline to flush blood out of the vascular system the brain was removed and measured, quickly dissected into two halves along the corpus callosum. One brain half was postfixed in 4% paraformaldehyde (PFA) for immune-histochemical staining used for our previous publication [10]. The other half of the brain was dissected into three parts (frontal, parietal and occipital), and the hippocampus was removed. In this study, the whole hemibrain was used, excluding the hippocampus. All parts were collected, a crude mixture was prepared, frozen in liquid nitrogen and stored at − 80 °C until further biochemical analysis.

### Preparation of Aβ-40

Aβ-40 and Aβ-42 are the predominant species in the Aβ family, with the former being more abundant and the latter more toxic. Because Aβ-40 contains the same metal binding amino residues (His-6, -14 and -13) as Aβ-42 and is less expensive and safer to work with and previously it was shown by Chen et al. [20] for proper in vitro aggregation capability, we chose Aβ-40 for our assays. Human Aβ-40 (Sigma Aldrich. Ltd., Germany, ∼ 90%) stock solutions were prepared according to the manufacturer instructions. Lyophilized Aβ-40 was dissolved in Ca^2+^-free Phosphate Buffer Saline at 1 mg/ml (230 µM) and incubated 37 °C for 4 days.

### Disaggregation assays

Disaggregation analysis of Aβ-40 was performed according to Chen et al. [20] with minor modifications. In disaggregation tests, Aβ-40 Zn^2+^/Cu^2+^ aggregates were prepared by treating fresh Aβ-40 solution with Zn^2+^ or Cu^2+^ and incubating at 37 °C for 24 h with constant agitation. Afterwards, DMSO-solved *Lactobacillus acidophilus* and *Bifidobacterium longum* lysate, FRAMELIM® and DMSO—as a vehicle control—was added and incubated at 37 °C for another 24 h with continuous agitation. The applied dose of *Lactobacillus acidophilus* and *Bifidobacterium longum* lysate was 10 µg/ml. In case of FRAMELIM® low (10 µg/ml) and high (100 µg/ml) dose was applied. The high dose of FRAMELIM® contained ∼ 10 µg/ml postbiotic equivalent. To characterize modulation of the aggregation UV/Vis spectrum (wavelength range of 190‒840 nm) was measured as well by DeNOVIX DS-11 FX Spectrophotometer/Fluorometer before ThT fluorescence assay as well.

### Fluorescence of thioflavin T (ThT)

After 2 days of disaggregation assay, DMSO-solved ThT at final concentration of 6 µM was applied to detect beta-amyloid aggregates. The fluorescence was measured after shaking 5 min in 100 µl volume of the incubated sample on ClarioStar plate reader (BMG Laboratories, Germany). Fluorescence spectrum was examined (λ_ex_ = 415 nm) with an exit slit width of 1 nm between 442 and 600 nm (ClarioStar, Germany). The sample compartment was kept at 21 °C.

### Mitochondrial, cytosolic and nuclear fraction preparation

Fractionation was performed according to Frezza et al. [21] with minor modifications. Briefly, the frozen brain tissue of each animal was immersed in ice-cold IB_c_ buffer (10 mM Tris-MOPS, 10 mM Tris-EGTA and 0.2 M Sucrose pH 7.4) in a ddH_2_O rinsed, precooled potter glassware, buffer volume was 10-fold. The tissue was homogenized by 3–4 times gentle stroke at 4 °C. The homogenate was centrifuged at 600×*g*, at 4 °C for 10 min. Supernatant was processed to further separation. The pellet included extracellular matrix elements, tissue debris and nuclear fraction was sonicated and sampled for Western blot. The supernatant was further centrifuged at 7000×*g*, at 4 °C for 10 min. The mitochondrial pellet was solved up in Ibc buffer and used for gelatine zymography and/or sampled to further Western blot analysis. The initial and leftover supernatant was processed to Western blot sample considering as total and cytosolic fraction. Protein concentration was measured in each fraction using the Bradford assay [22].

### Analysis of mitochondrial proteolytic activity analysis through gelatine zymography

Zymography assays were performed according to Kupai et al. [23] with minor alterations. Briefly, the zymography was performed using a 10% SDS-PAGE separation gel with 0.1% of gelatine. Forty micrograms of mitochondria fraction or total lysate from each group was incubated on charging buffer (100 mM Tris pH 6.8, 5% SDS, 20% glycerol, 0.1% bromophenol blue) for 10 min on ice, in a proportion of 1:1 (v/v). After the run, the gels were incubated in renaturation buffer (2.5% Triton X-100) for 30 min, with soft agitation. Then, the zymogram gels were changed to a development buffer (50 mM Tris, 5 mM NaCl, 10 mM CaCl_2_, 1 µM ZnCl_2_, 0.02% (v/v) Triton X-100, pH 7.4) for more 30 min, also with soft agitation. Finally, the gel immersed into a new development buffer, and incubated overnight at 37 °C, in case of mitochondrial proteins the incubation time was 40 h. For specific inhibition studies zymograms were incubated in the presence of 10 mM EDTA, 10 mM EGTA or 6,4 µM CDDO. The zymography gels were stained with 0.12% (w/v) Coomassie Blue G-250 prepared in 20% methanol, after 1 h fixation in a solution of 10% acetic acid and 40% methanol. Distaining was performed with 25% methanol and stained gels were scanned.

### Western blot analysis

The brain tissue of each animal was homogenized in ice and lysed in a lysis buffer containing 137 mM NaCl, 20 mM Tris-HCl pH 8.0, 1% Nonidet P-40, 10% glycerol, and tablets of protease and phosphatase inhibitors. Lysates were centrifuged for 15 min at 14,000×*g* at 4 C. Protein concentration was measured using the Bradford assay [22]. Proteins were separated on 8–15% (v/v) SDS-PAGE (sodium dodecyl sulphate-polyacrylamide) gels at room temperature (RT) and transferred onto PVDF membrane (pore size: 0.2 and 0.4 μm) at 4 °C. The nonspecific binding of immune-proteins was blocked with 5% BSA (bovine serum albumin) dissolved in Tris-buffered saline, Tween-20 (TBS-T) for 1 h at RT. After blocking, the membranes were incubated with primary mouse and human reactive APP rabbit (1:3000, Abcam-ab101492) and human reactive APP mouse (1:3000, Biolegend-6E10 clone); LONP1 mouse (1:3000, Proteintech-66043-1-Ig), SDHA rabbit (1:3000, SantaCruz-sc-98253), NF-kB rabbit (1:3000, Cell Signaling-#3032), GAPDH (mouse, 1:40,000; Sigma-Aldrich) antibodies in TBS-T containing 5% BSA, overnight at 4 C. After overnight incubation the membranes were rinsed in TBS-T attended by 1 h of incubation with HRP-conjugated secondary antibodies at RT. The secondary HRP-conjugated antibodies were anti-rabbit, anti-mouse IgG in TBS-T containing 1% BSA (1:10,000; Jackson Immunoresearch). Between incubation times, the membranes were washed repeatedly—4 times for 10 min—and after the last washing session, incubated with an enhanced chemiluminescent reagent (ECL Star Enhanced Chemiluminescent Substrate; Euroclone) for 1 min. The protein bands were visualized on X-ray film. Bands were quantified by ImageJ 1.52 software, and total protein normalization was used for the analysis.

### Quantitative real-time PCR

Total RNA was extracted from hemi-brain tissue samples by using Nucleo Spin RNA plus RNA isolation kit (Macherey Nagel, Düren, Germany). RNA integrity was tested on ethidium bromide stained 2% agarose gel. RNA purity was measured with DeNovix ds-11 Spectrophotometer (DeNovix, Wilmington, USA) and samples with 260/280 nm absorbance higher than 2 were considered pure and processed forward. RT-cDNA synthesis reaction was performed by Tetro cDNA Synthesis Kit (Bioline London, UK) the total input RNA was 5 µg/reaction, with random hexamer primer amplification according to the manufacture’s protocol. Synthetized cDNA was 10 diluted with PCR grade DEPC-treated water and used for PCR amplification. The mRNA level was PCR quantified by using Corbett Research RG-6000 Real Time PCR Thermocycler (QUIAGEN, USA). IMMomix mastermix (Bioline London, UK) was used. Reverse and forward primers were present at 1 µM concentration with 1% Evagreen. Primer efficiency was accepted at E = 0.90–1.1 respectively, the amplification specificity was checked by melting point analysis. The thermal profile was 50 °C for 3 min, 95 °C for 10 min, and subsequently 40 cycles of 95 °C for 20 s, 60 °C for 20 s and 72 °C for 20 s. PCR amplification were performed using the following primers: for mouse APP, forward 5′-TGTGCCAGCCAATACCGAA-3′ and reverse 5′-CCAGAACCTGGTCGAGTGGT-3′; for mouse NF-κB, forward 5′-ATGCCGAACTTCTCGGACAG-3′ and reverse 5′-GTGTTTATGGTGCCATGGGTG-3′; for SOD-1, forward 5′-GGAACCATCCACTTCGAG-3′ and reverse 5′-CTGCACTGGTACAGCCTTGT-3′; for mouse β-actin, forward 5′-AGATCAAGATCATTGCTCCTCCT-3′ and reverse 5′-ACGCAGCTCAGTAACAGT-3′. The primer sequences shown above were synthesized by Sigma Aldrich (Germany). Results were analysed using the software with relative quantification method and β-Actin as endogenous control was used. Fold changes in each target mRNA expression relative to β-Actin were calculated by Pfaff method and the 2^−ΔΔCT^ method. Expression of mRNA is defined as the change in mRNA copy numbers relative to APP/PS1 control samples.

### Statistical analysis

A priori sample size were estimated by G*power program (version 3.1.9.7.) parameters were set 0.05 for α and 0.8 for β, inputs for calculations are used from our previously published study [10]. We employed Prism 5.01 for Windows (GraphPad, United States) software for statistical and graphing analysis. Two-way analysis of variance (ANOVA) with exercise and postbiotic treatment as main factors was used for evaluation of differences among APP/PS1 groups. Bonferroni’s Post-hoc test was conducted to further interrogate statistically significant ANOVA results. Unpaired Student T-test was used comparing wild type and APP/PS1 Control groups to test the effect of the human transgene. Statistical significance was considered if P < 0.05. All values are reported as means ± SEM.

## Results

### Postbiotic supplement disintegrates β-sheet aggregation of Aβ-40

**Fig. 1:**
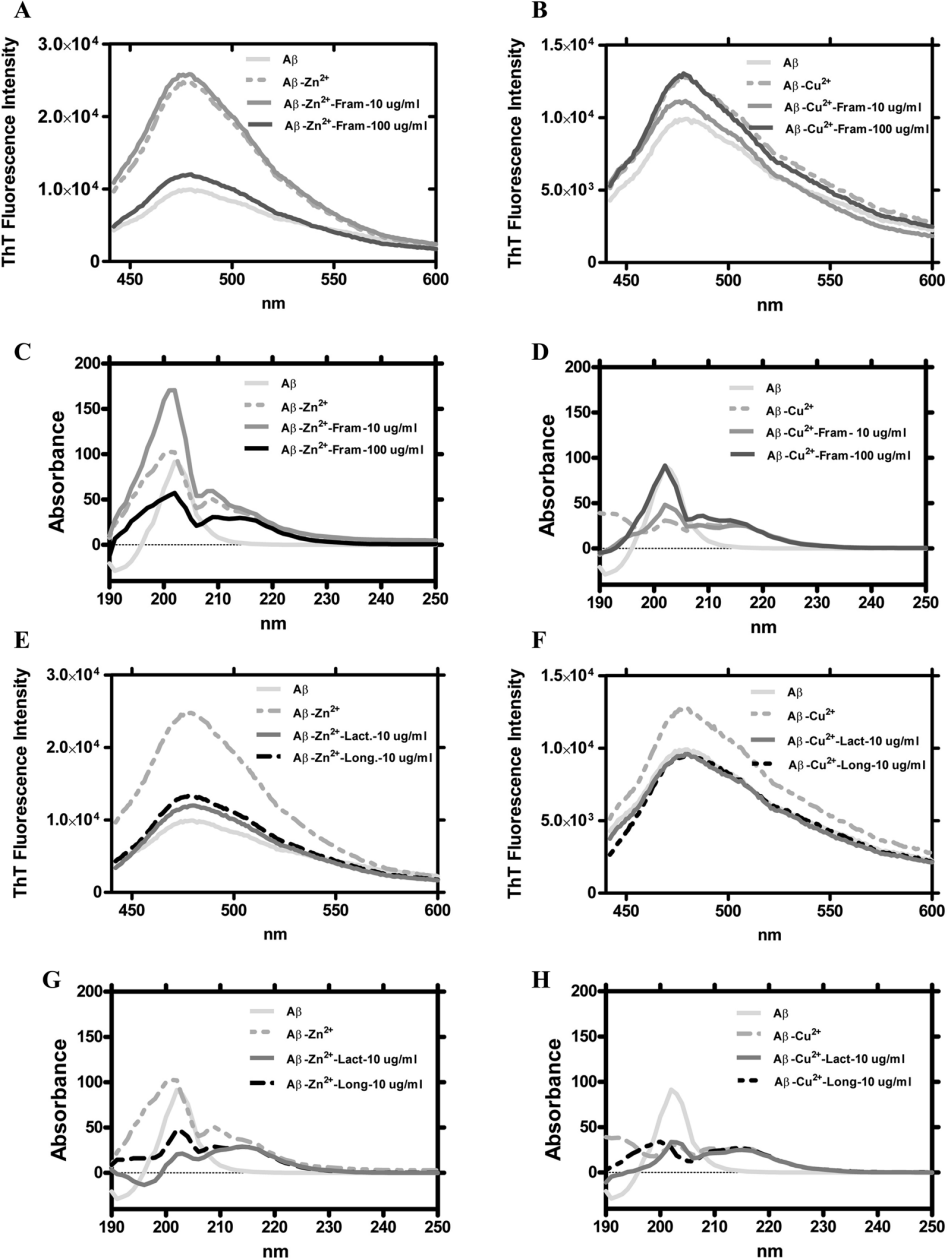
Postbiotics inhibited Cu^2+^ and Zn^2+^ induced aggregation of Aβ-40. The disaggregation assay began with incubation of Aβ-40 (40 µM) with metal ions ([Cu^2+^] = 25 µM; [Zn^2+^] = 25 µM) at 37 °C for 1 day with gentle shaking, then appropriate postbiotics were added at the above doses: ([FRAMELIM®] = 10 and 100 µg/ml; [*Bifidobacterium longum lys*.] = 10 µg/ml; [*Lactobacillus acidophilus lys.*] = 10 µg/ml). After 1 day of further agitation, both UV/Vis spectral analysis and ThT fluorescence assay was performed. **A** FRAMELIM® inhibited Zn^2+^-induced aggregation only at dose of 100 µg/ml, whereas **B** Cu^2+^ induced increase in ThT fluorescence was much smaller compared to the effects of Zn^2+^ ions indicating limited aggregation. Cu^2+^ enhanced β-sheet aggregation was only slightly altered by FRAMELIM®. **C** FRAMELIM® at both dosages basically modulated the absorption pattern of Zn^2+^-induced Aβ-40 aggregates. **D** For Cu^2+^-induced Aβ-40 aggregation a minor modulation in the absorbance in the range of 190–200 nm by FRAMELIM® is observed. **E** Bifidobacterium *longum lys.* and *Lactobacillus acidophilus lys.* strongly reduced aggregations at dose of 10 µg/ml in the presence of Zn^2+^ ions. **F** Similar results were observed in the case of Cu^2+^ ions: both postbiotics were very effective in inhibition. **G** Both postbiotics basically modulated absorbance pattern of Zn^2+^-induced Aβ-40 aggregates. **H** For Cu^2+^-induced Aβ-40 aggregation, a slight modulation of absorption in the 190–200 nm range by postbiotics is observed, similar to FRAMELIM®

First, we investigated whether postbiotic supplementation can influence the formation of Aβ-40 plaques. We performed a disaggregation assay to characterize Aβ-40 plaque formation in the presence of metal ions and postbiotics. The intensity of Thioflavine (ThT) fluorescence measured the beta-sheet content of Aβ-40 plaque. It increased significantly in the presence of Zn^2+^, whereas the same dose of Cu^2+^ ions resulted in slight increase in fluorescence. This suggests that Zn^2+^ promotes the formation of Aβ-40 plaque (Fig. 1A, B). In the presence of Zn^2+^ and Cu^2+^ ions, characteristic changes in UV/Vis spectra from 190 to 230 nm were also observed. The presence of Zn^2+^ ions increased the Aβ-40 absorbance outside the range of 200 to 210 nm (Fig. 1C). Interestingly, presence of Cu^2+^ ion decreased the Aβ-40 absorbance in the range of 200 to 210 nm and increased it outside this range (Fig. 1D). Based on ThT fluorescence, only at high 100 µg/ml dose FRAMELIM® inhibit Zn^2+^ ion induced formation of Aβ-40 plaque (Fig. 1A) while the aggregation promoted by Cu^2+^ was only slightly modulated (Fig. 1B). Based on UV/Vis spectra, FRAMELIM® inhibited the increase in Aβ-40 plaque absorbance caused by Zn^2+^ ions only at high doses (Fig. 1C). However, the reduction of Aβ-40 absorbance by Cu^2+^ ion in the range of 200 to 210 nm was compensated by FRAMELIM® (Fig. 1D). ThT fluorescence also showed that both *Bifidobacterium longum* and *Lactobacillus acidophilus* lysates effectively reduced Aβ-40 plaque formation enhanced by Cu^2+^ or Zn^2+^ ions (Fig. 1E, F). These changes were also reflected in the modulation of UV/Vis spectra: the increase in Aβ-40 absorbance induced by Zn^2+^ ion was spectacularly reduced by both postbiotics (Fig. 1G). However, the modulation of the UV/Vis spectra of Aβ-40 in the case of Cu^2+^ ions was only slightly affected by the postbiotics (Fig. 1H). Overall, this suggests that the postbiotics used may be an effective component of FRAMELIM® in reducing amyloid-ß plaques also in vivo*.*

### Postbiotic supplementation decreased human transgene APP-level

**Fig. 2:**
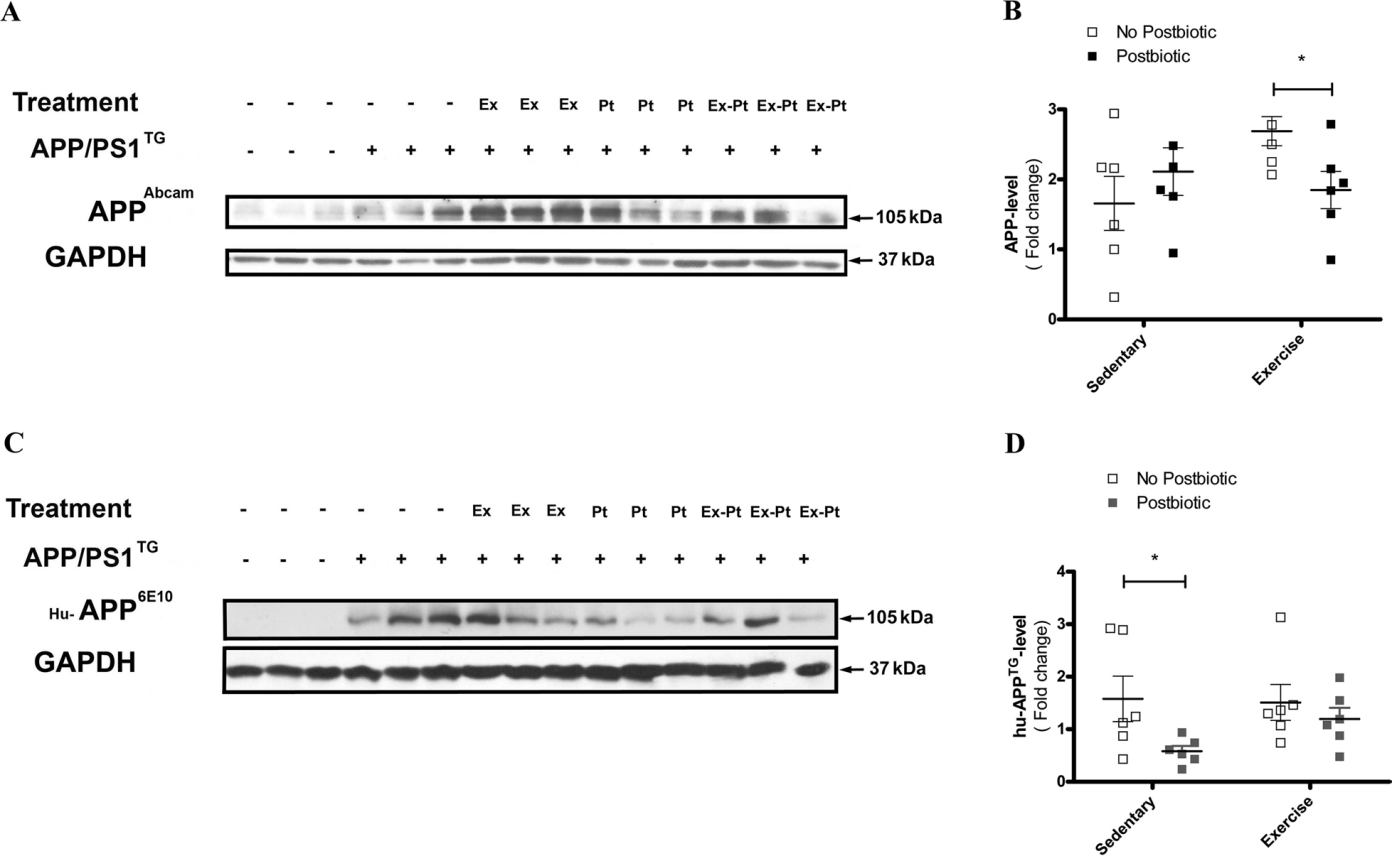
Effects of exercise training and postbiotic treatment on APP levels (**A**) mouse endogenous APP/PS1 levels were determined by Western blotting with the APP-1 antibody (Abcam; ab101492) in the brain homogenates of wild type mice (Wt), control mice (APP/PS1^TG^), exercise trained mice (APP/PS1^TG^-Ex), mice with postbiotic treatment (APP/PS1^TG^-Pt), mice with combined training and postbiotic supplementation (APP/PS1^TG^-Ex-Pt) mice. **C** Human APP^TG^ levels were detected with the human reactive anti-β-Amyloid antibody (Biolegend; clone 6E10). **B**, **D** Quantitative analysis of optical densities for mouse and human APP1 expression. Results show means ± SEM (n = 6). *P < 0.05 compared with APP/PS1 groups, determined by analysis of variance using Two-way ANOVA, followed by Bonferroni’s post-hoc test. Representative Western blots are shown in **A** and **C**

Because postbiotics chelate metal ions, it is plausible that they can affect full-length human APP^TG^ expression and transcription. Therefore we measured both endogenous mouse and human APP levels by Western blot (Fig. 2A, D).

The endogenous mouse level was significantly increased in APP/PS1TG mice: Wt vs. APP/PS1 control groups, 0.33 ± 0.07 vs. 1.66 ± 0.39; *P < 0.05 unpaired Student T-test. (Fig. 2A). Two-way test ANOVA followed by post-hoc test revealed that the main effect of interaction between postbiotic treatment and exercise was significant [F(1, 20) = 4.437, P < 0.05], indicating that postbiotic treatment has a decreasing effect on exercise-induced increase in endogenous APP-level (Fig. 2A, B).

Analysis with human reactive anti-amyloid-β antibodies (clone 6E10) showed that the APP/PS1 transgene markedly increased human APP^TG^ levels in all APP/PS1 groups examined, whereas it did not in Wt group, as expected. Postbiotic treatment decreased human APP^TG^ levels, but exercise training after postbiotic treatment neutralized the observed beneficial decrease revealed by two-way analysis ANOVA. A significant main effect of postbiotic treatment [F(1, 20) = 4.741, P < 0.05] was observed. However, Bonferroni post-hoc analysis revealed no significant changes. (*P < 0.05; Fig. 2C, D).

### Postbiotic supplementation and exercise training modulate expressions of AD-related genes

**Fig. 3:**
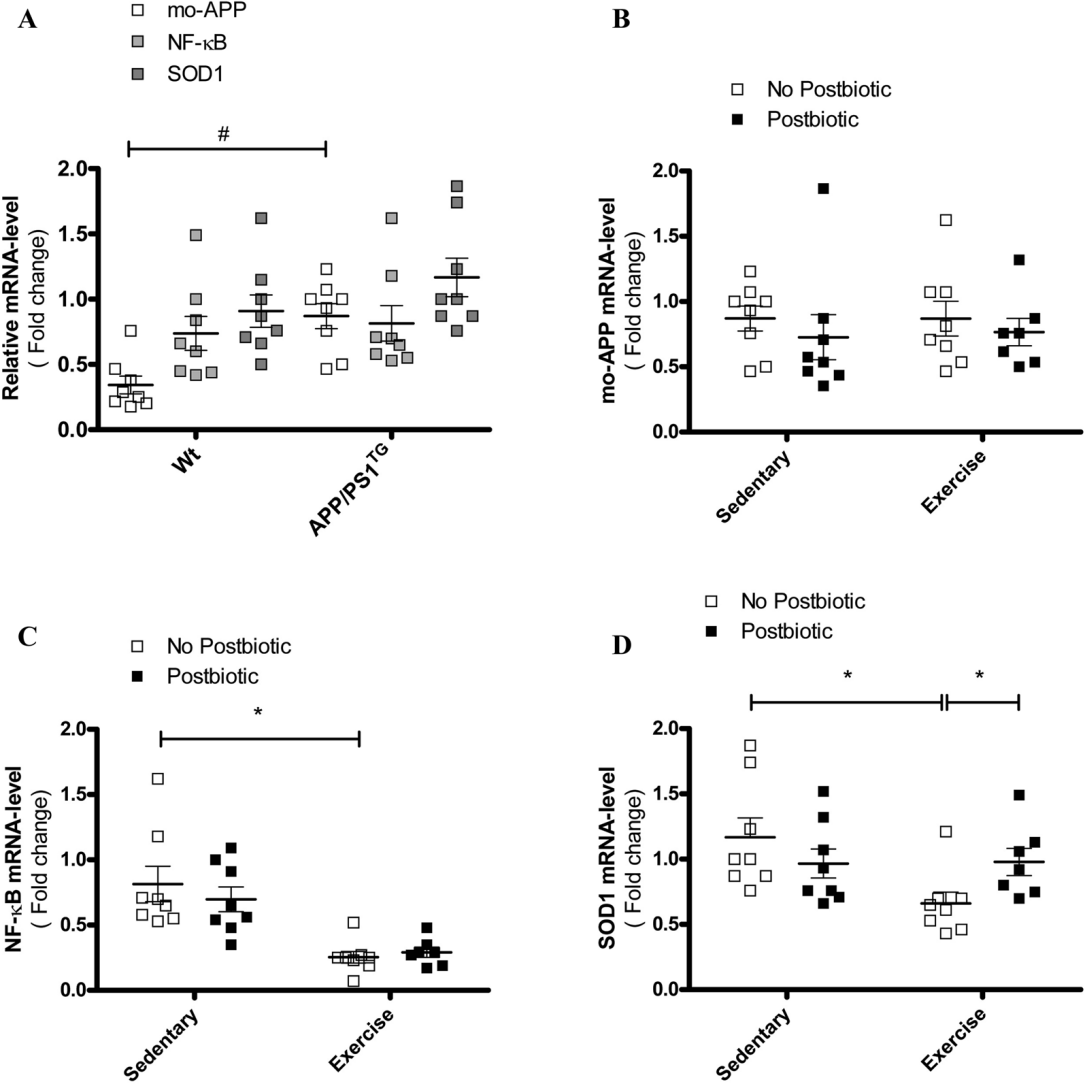
Evaluation of APP, NF-kB, SOD-1 mRNA levels followed by exercise training and/or postbiotic supplementation. 2^−ΔΔCt^ method used to relative quantification of gene expression in the hemibrain homogenates of wild type (Wt), control (APP/PS1^TG^), exercise trained (APP/PS1^TG^-Ex), postbiotic supplemented (APP/PS1^TG^-Pt), and combined (exercise trained and postbiotic supplemented) (APP/PS1^TG^-Ex-Pt) mice for **B** mouse APP, **C** NF-kB and **D** SOD1 gene expression. Comparison between Wt and APP/PS1^TG^ for expressions of all the genes is presented as well (**A**). The results showed the means ± SEM (n = 7–8). ^#^P<0.05 compared with the APP/PS1 Control group vs Wild type group, as determined by analysis of variance by unpaired Student T-test. *P < 0.05 compared with the APP/PS1 groups, as determined by analysis of variance by Two-way ANOVA, followed by Bonferroni’s post-hoc test

Because inflammation-induced oxidative stress plays a critical role in the development of AD, we examined the mRNA levels of several relevant transcription factors and proteins. The presence of the APP/PS1 transgene resulted in increased mouse APP mRNA-level in all APP/PS1 groups examined (*P < 0.05; Fig. 3A) but no significant changes were found between groups (Fig. 3B). We did not detect any significant changes in NF-κB expression between the wild type and APP/PS1 control group (Fig. 3A). However, as shown in the quantitative summary, exercise training significantly decreased NF-κB expression compared with the sedentary groups [two-way ANOVA followed by post-hoc test. F(1, 27 = 28.39, P < 0.0001 for main effects of exercise training (*P < 0.0001; Fig. 3C). The SOD1 expression level between the wild type and APP/PS1 control group was not significantly changed (Fig. 3A), but exercise training significantly decreased SOD1 expression compared with the sedentary group [two-way ANOVA followed by post-hoc test. F(1, 27 = 4.56, P < 0.05 for main effects of exercise (*P < 0.05; Fig. 3D). Surprisingly, postbiotic treatment significantly invalidated the effect of exercise treatment [two-way ANOVA followed by post-hoc test. F(1, 27 = 5.03, P < 0.05 for the main effect of exercise × postbiotic treatment interaction (*P < 0.05; Fig. 3D).

### Postbiotic supplementation and exercise training elevated mitochondrial level of LONP1 and SDHA but level of NDUFV1 showed no alteration

**Fig. 4:**
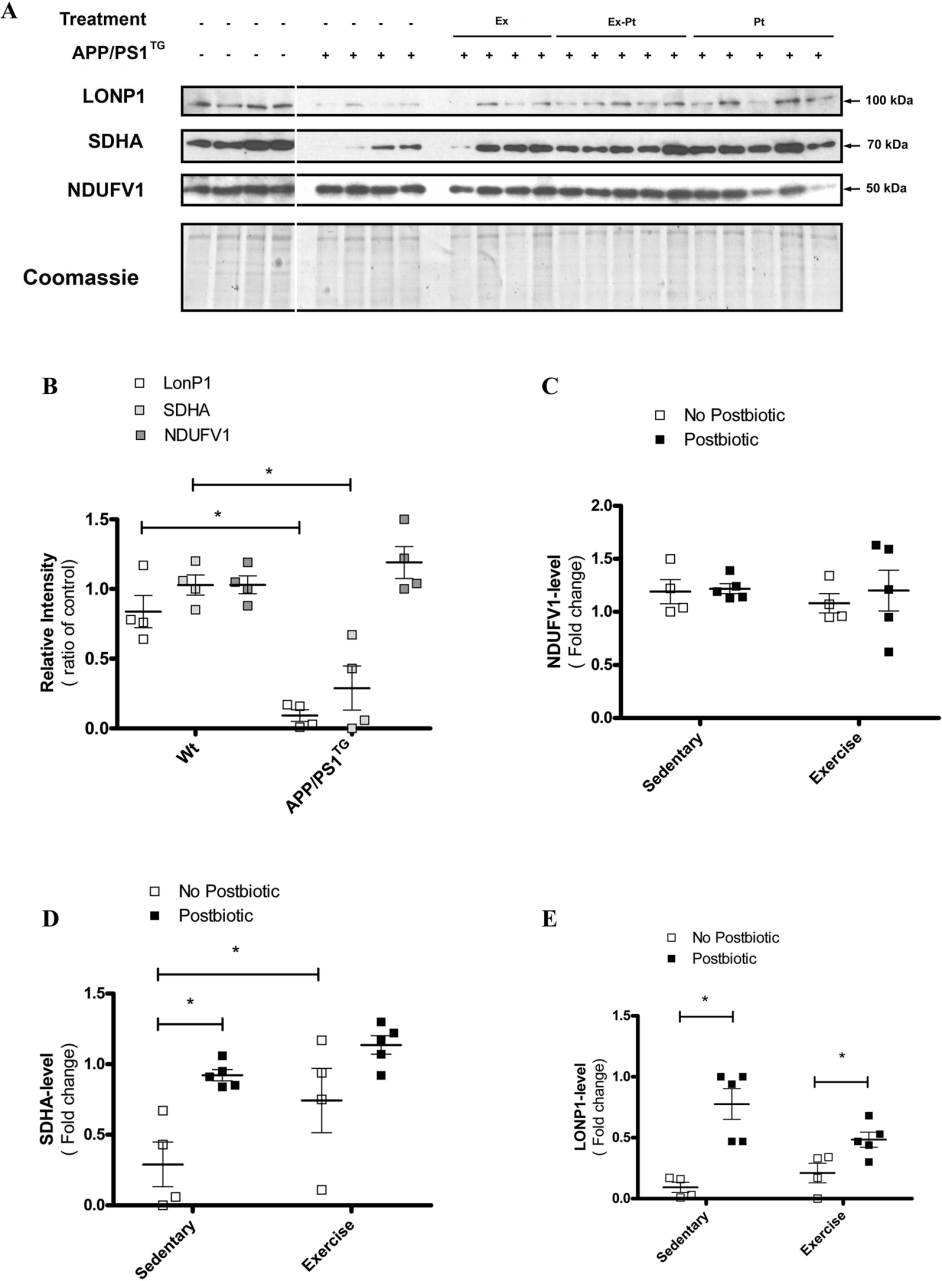
Exercise training and/or postbiotic treatment alter mitochondrial composition. **A** Mitochondrial LONP1, SDHA and NDUFV1 levels were determined by Western blotting in the mitochondrial fraction of the brain of wild type (Wt), control (APP/PS1^TG^), trained (APP/PS1^TG^-Ex), postbiotic supplemented (APP/PS1^TG^-Pt) and combined (trained and postbiotic-supplemented) (APP/PS1^TG^-Ex-Pt) mice. Quantitative analysis of optical densities of protein bands for **B**, **C** NDUFV1, **B**, **D** SDHA and **B**, **E** LONP1 expression. Data were normalized to total protein levels determined by Coomassie staining of proteins transferred to the membrane. Bands are elements of the same representative gel, the continuity of the gel was broken, and the irrelevant group was omitted, indicated by white area. Western blot results show means ± SEM (n = 4–5). ^#^P < 0.05 compared with APP/PS1 control group vs wild type group, determined by analysis of variance using unpaired Student T-test. *P < 0.05 compared with APP/PS1 groups, determined by analysis of variance using Two-way ANOVA, followed by Bonferroni’s post-hoc test

We examined the mitochondrial level of the mitochondrial matrix protein LONP1, mitochondrial inner membrane protein NDUFV1, and SDHA, which is externally anchored to the succinate dehydrogenase complex. Analysis of mitochondrial proteins showed that the presence of APP/PS1 transgene leads to a fundamental change in mitochondrial protein composition: LONP1 and SDHA levels decreased significantly, whereas NDUFV1levels showed no change (Fig. 4A, B). The level of NDUFV1, a hydrophilic protein tightly bound to the mitochondrial inner membrane, remained constant in all groups. Coomassie staining of the PVDF membrane proved uniform loading of mitochondrial proteins (Fig. 4A–C). Postbiotic treatment and exercise training restored mitochondrial SDHA levels [two-way ANOVA followed by post-hoc test. F(1, 14) = 15.72, P = 0.0014; and F(1, 14) = 6.64, P < 0.05; for main effects of postbiotic treatment and exercise training, P < 0.01 for sedentary vs. postbiotic post-hoc test] (Fig. 4A, D). Postbiotic treatment restored matrix protein, and LONP1 levels [two-way ANOVA followed by post-hoc test. F(1, 14) = 29.15, P < 0.0001; and F(1, 14) = 5.33, P < 0.05; for main effects of postbiotic treatment and postbiotic × exercise interaction, P < 0.001 for sedentary vs. postbiotic post-hoc test] (Fig. 4A, E). The change in LONP1 protein levels and activity do not always correlate, because LONP1 activity is post-translationally regulated [24].

**Fig. 5:**
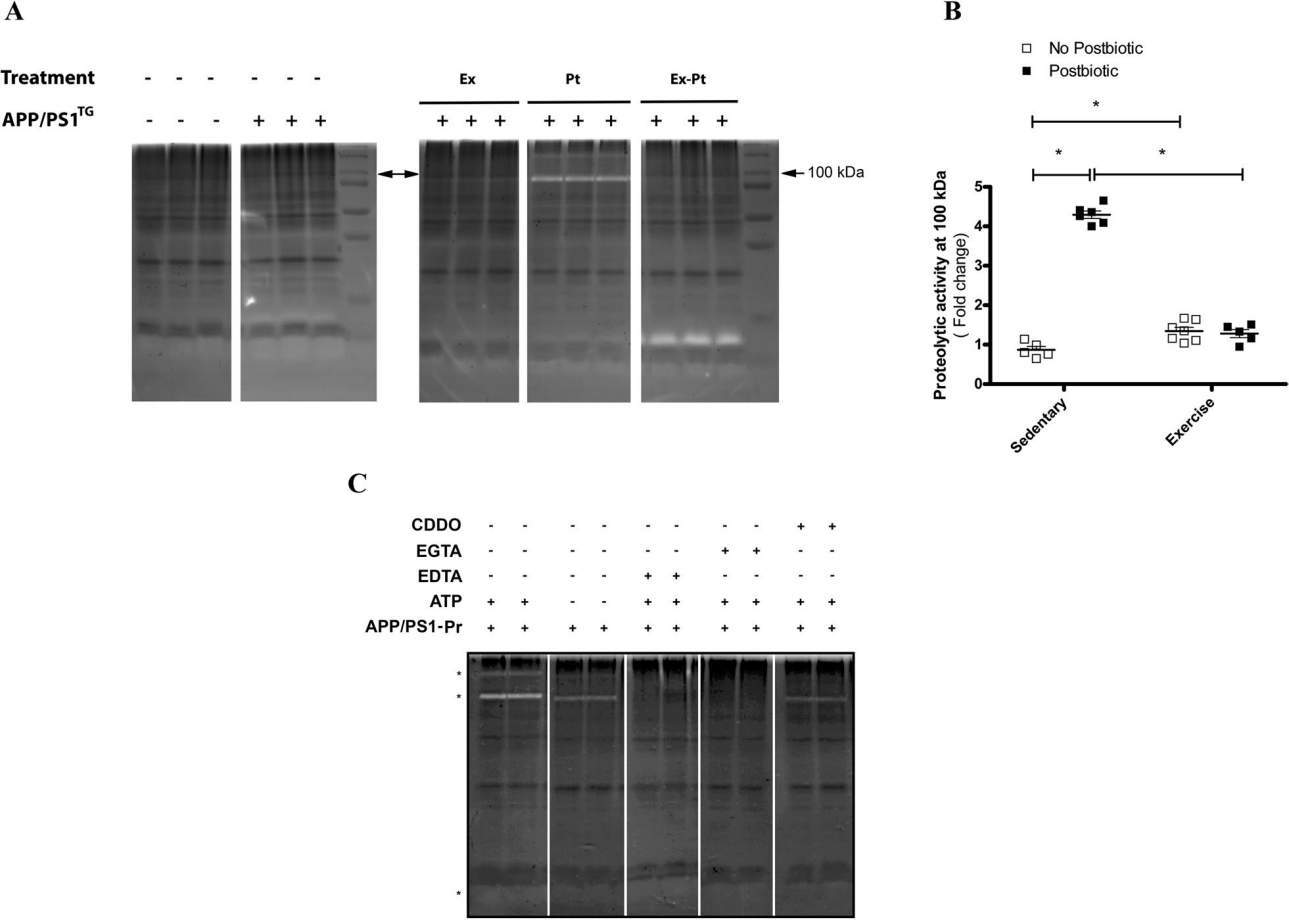
Postbiotic treatment increases mitochondrial LONP1 activity. **A** Representative zymography of the mitochondrial fraction of the brain of wild type (Wt), control (APP/PS1^TG^), trained (APP/PS1^TG^-Ex), postbiotic-supplemented (APP/PS1^TG^-Pt) and combined (trained and postbiotic-supplemented) (APP/PS1^TG^-Ex-Pt) mice. The major gelatinase activity was found at ∼ 100 kDa. Bands from the groups were from two separate gels developed at the same time and under same conditions using the same standards and molecular weight markers to ensure quality control. Three elements of the groups were excised and separated by blank space. **B** Semi-quantitative optical density analysis of proteolytic activity for the band at 100 kDa. Data were normalized to undigested area of protein load near the 100 kDa band in each individual sample. Zymography results show means ± SEM (n = 5–6). *P < 0.05 compared with groups APP/PS1, determined by analysis of variance with Two-way ANOVA, followed by Bonferroni’s post-hoc test. **C** Gelatinase activity of the postbiotic-supplemented sample (APP/PS1^TG^-Pt) was further characterised. The activity was dependent on both ATP and Ca^2+^ and Mg^2+^ ion chelators, suggesting ATP-dependent metalloproteinase activity. The specific LONP1 inhibitor, CDDO, inhibited almost all activities. Two elements of the groups were excised and separated by white space

For this reason, we also performed an analysis of proteolytic activity using gelatine zymography [25]. It was found that postbiotic supplementation resulted in some gelatinase activities. Slight bands are observed at ∼ 250 and ∼ 150 kDa, and defined bands at ∼ 100 and ∼ 18 kDa. The maximum activity is 100 kDa, which corresponds to the estimated molecular weight of monomer LONP1 (black asterisks, Fig. 5A). Postbiotic treatment resulted in a strong increase in activity compared with the sedentary control [two-way ANOVA followed by post-hoc test. F(1, 19) = 298.18, P < 0.0001; and F(1, 19) = 320.88, P < 0.0001; for main effects of postbiotic treatment and postbiotic × exercise interaction, P < 0.001 for sedentary vs. postbiotic post-hoc test] (Fig. 5A, B). Exercise training resulted in a less pronounced but still significant increase in gelatinase activities at ∼100 kDa [two-way ANOVA followed by post-hoc test. F(1, 19) = 170, P < 0.0001 for main effects of exercise training] (Fig. 5A, B). Interestingly, concurrent treatment did not resultin any synergism in gelatinase activity, suggesting that exercise training somehow unmasked the postbiotic-induced tremendous LONP1 activity (Fig. 5A, B). To prove that the observed postbiotic treatment induced gelatinase activities were solely due to LONP1 activity, some important factors of the activity were examined. The activity was inhibited by ATP deprivation, by chelation of Ca^2+^ and Mg^2+^ ions with EGTA and EDTA, by specific LONP1 inhibitor CDDO, resulting in significant decrease in activity (Fig. 5C). These data suggest that postbiotic treatment resulted not only in increased mitochondrial levels but also in activation of LONP1.

## Discussion

There is a tremendous need for natural and pharmacological interventions that can prevent, attenuate, or slow the progression of AD. Here we report that both *Bifidobacterium longum* and *Lactobacillus acidophilus* lysates effectively reduce Cu^2+^ or Zn^2+^ induced aggregation of Aβ-40 in vitro and in vivo models. It has been suggested metal ions, Cu^2+^, and Zn^2+^ play a crucial role in altering the chemical architecture of Aβ-40/42 in AD and increasing its toxicity [26–29]. Cu^2+^ and Zn^2+^ can hypermetalize Aβ-40/42, leading to production of H_2_O_2_ and oxidative stress [30]. In fact, it has been suggested that the increased incidence of AD in women may be at least partly dependent on the higher activity of Zn transporter ZnT3 and the suppressed ability to chelate metal ions [30]. We have previously shown that supplementation of FRAMELIM® with *Bifidobacterium longum* and *Lactobacillus acidophilus* can reduce the accumulation of Aβ−40 aggregates and reduce toxicity [10], and here we have shown that this was mediated, at least in part, by chelation of Cu^2+^, and Zn^2+^.

The postbiotic treatment can reduce the levels of APP^TG^, which leads to a decrease in the formation of terminal Aβ-40 and Aβ-42. These peptides not only accumulate outside cells but also invade mitochondria and cause mitochondrial dysfunction [31]. By reducing the presence of the peptides in mitochondria, postbiotic treatment may also help in reducing mitochondrial dysfunction. Inflammation is a concomitant and/or causative process of AD [32–34]. Transcription factors such as SP1 and NF-kB are mediators of inflammatory processes, and downregulation of these transcription factors not only suppresses inflammation but also decreases reactive oxygen species production (ROS) [4]. Here, we confirmed that exercise especially in conjunction with postbiotic treatment reduces the involvement of NF-kB [35, 36] in the inflammatory process, and our data showed that this process plays a neuroprotective role in APP/PS1 model [10].

We have previously shown that the mitochondrial housekeeping enzyme, LONP1 is sensitive to exercise stimuli and that exercise induction is associated with reduced accumulation of damaged mitochondrial proteins in skeletal muscle and increased endurance performance [37–39]. Our data demonstrate that physical training and postbiotic treatments prevent the loss of mitochondrial matrix proteins. The treatments increased LONP1 levels and activity as determined by gelatine zymography. LONP1 is responsible for the removal of damaged proteins in mitochondria, and plays a critical roles in cell survival because of the excessive generation of ROS in this organelle. AD is known to be associated with increased oxidative stress [40, 41], thus the present new observation, that exercise and postbiotic treatments increase LONP-1 levels and activity adds an important new mosaic to the better understanding of exercise and postbiotic treatments at AD.

It should be noted that our study has limitations, as it only considers the early and medium-term changes associated with the progression of Alzheimer’s disease in mice. In addition, functional mitochondrial assays beyond LONP1 activity measurement are necessary to outline the context of improved mitochondrial function.

## Conclusion

The results of the present study demonstrate that postbiotic treatments and exercise training have beneficial effects on the APP/PS1^TG^ mouse model of Alzheimer’s disease, mediated in part by the chelating effects of heat-inactivated *Bifidobacterium longum* and *Lactobacillus acidophilus* on Zn^2+^ and Cu^2+^ ions. In addition, the combined effects of exercise and postbiotics improve the antioxidant system, suppress inflammation, and mitochondrial homeostasis via activation of LONP1. These results give a hint that by mild training protocol in combination with the above postbiotics may offer more effective preventive treatment protocol for AD.

## Data Availability

All data generated or analysed during this study are included in this published article.
